# Supplementary material for: Disability and educational outcomes in Nepal: Evidence from the 2019 Multiple Indicator Cluster Survey
Source: PLOS Glob Public Health. 2026 Jun 4;6(6):e0006579. doi: 10.1371/journal.pgph.0006579 (PMC13235914; doi:10.1371/journal.pgph.0006579)
Supplement: S1 Table — Note: Values represent regression coefficients with standard errors in parentheses. Models include interaction terms between disability and gender (Panel A) and between disability and area of residence (Panel B). All models control for age group, household characteristics, maternal education, and household wealth. Reference categories are male for gender and urban for residence. * p < 0.10, ** p < 0.05, *** p < 0.01. (DOCX) [file pgph.0006579.s001.docx]

| **Panel A: Interaction between disability and gender** | | | | |
| --- | --- | --- | --- | --- |
| **Variables** | **Ever Attended School / Program** | **Highest Grade Attainment** | **Grade-For-Age Alignment** | **Difficulties adjusting to Routine** |
| Disability | -0.031*** (0.007) | -0.269***  (0.086) | -0.028  (0.022) | 0.146***  (0.016) |
| Female | -0.004  (0.004) | 0.133***  (0.046) | 0.043***  (0.012) | -0.006***  (0.009) |
| Disability × Female | 0.010  (0.010) | 0.142  (0.126) | 0.052  (0.033) | -0.020  (0.024) |
| **Panel B: Interaction between disability and area of residence** | | | | |
| **Variables** | **Ever Attended School / Program** | **Highest Grade Attainment** | **Grade-For-Age Alignment** | **Difficulties adjusting to Routine** |
| Disability | -0.023*** (0.006) | -0.261***  (0.084) | -0.010  (0.022) | 0.135***  (0.016) |
| Rural residence | -0.001  (0.004) | 0.059  (0.047) | 0.024***  (0.012) | -0.028***  (0.009) |
| Disability × Rural residence | -0.007  (0.010) | 0.134  (0.126) | 0.015  (0.033) | 0.004  (0.024) |
| Observations | 7,789 | 7,617 | 7,185 | 7,789 |
