# Supplementary figures and images for: Disability and educational outcomes in Nepal: Evidence from the 2019 Multiple Indicator Cluster Survey
Source: PLOS Glob Public Health. 2026 Jun 4;6(6):e0006579. doi: 10.1371/journal.pgph.0006579 (PMC13235914; doi:10.1371/journal.pgph.0006579)

## Panel A. By Gender

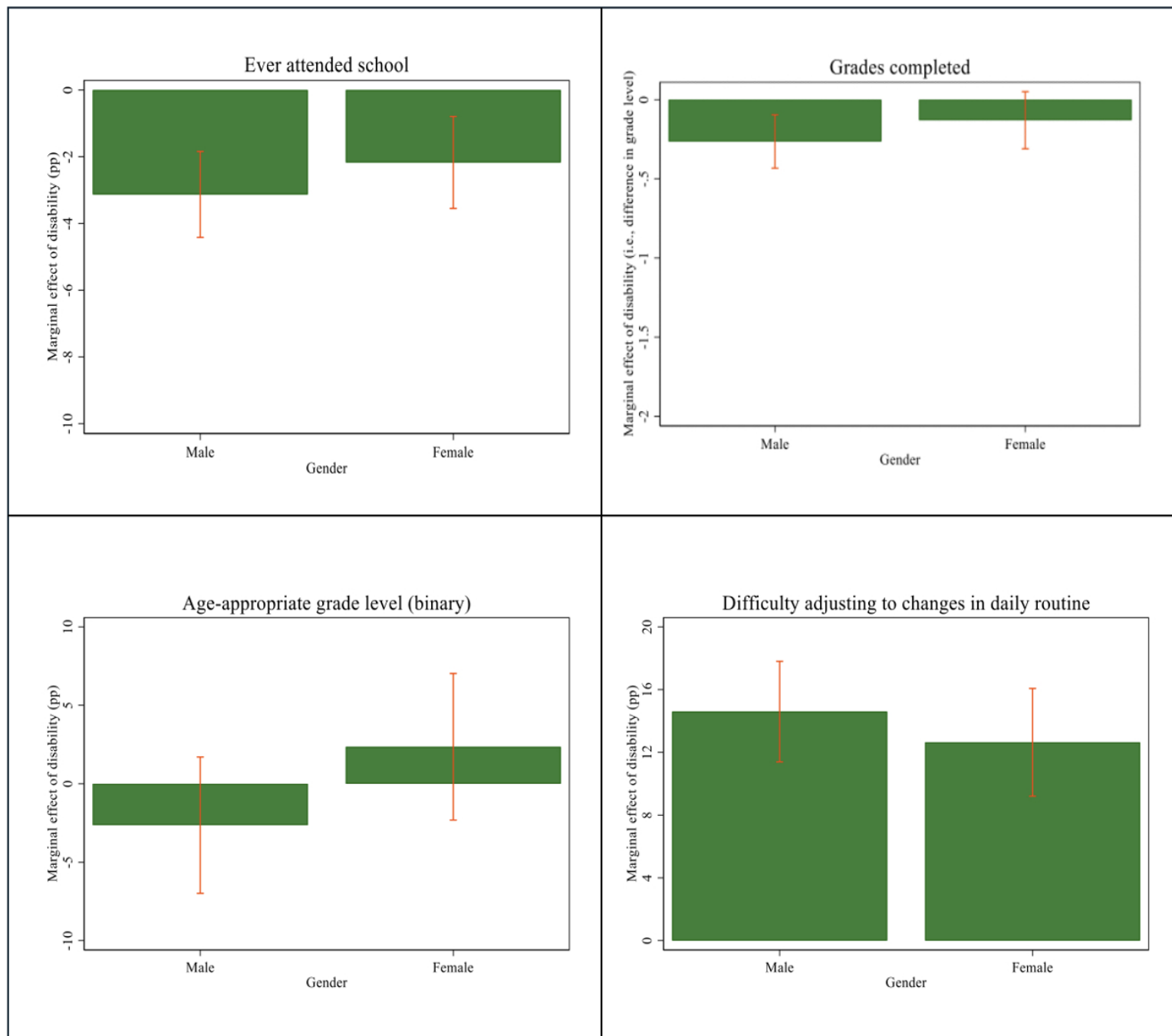

## Panel B. By Area of Residence

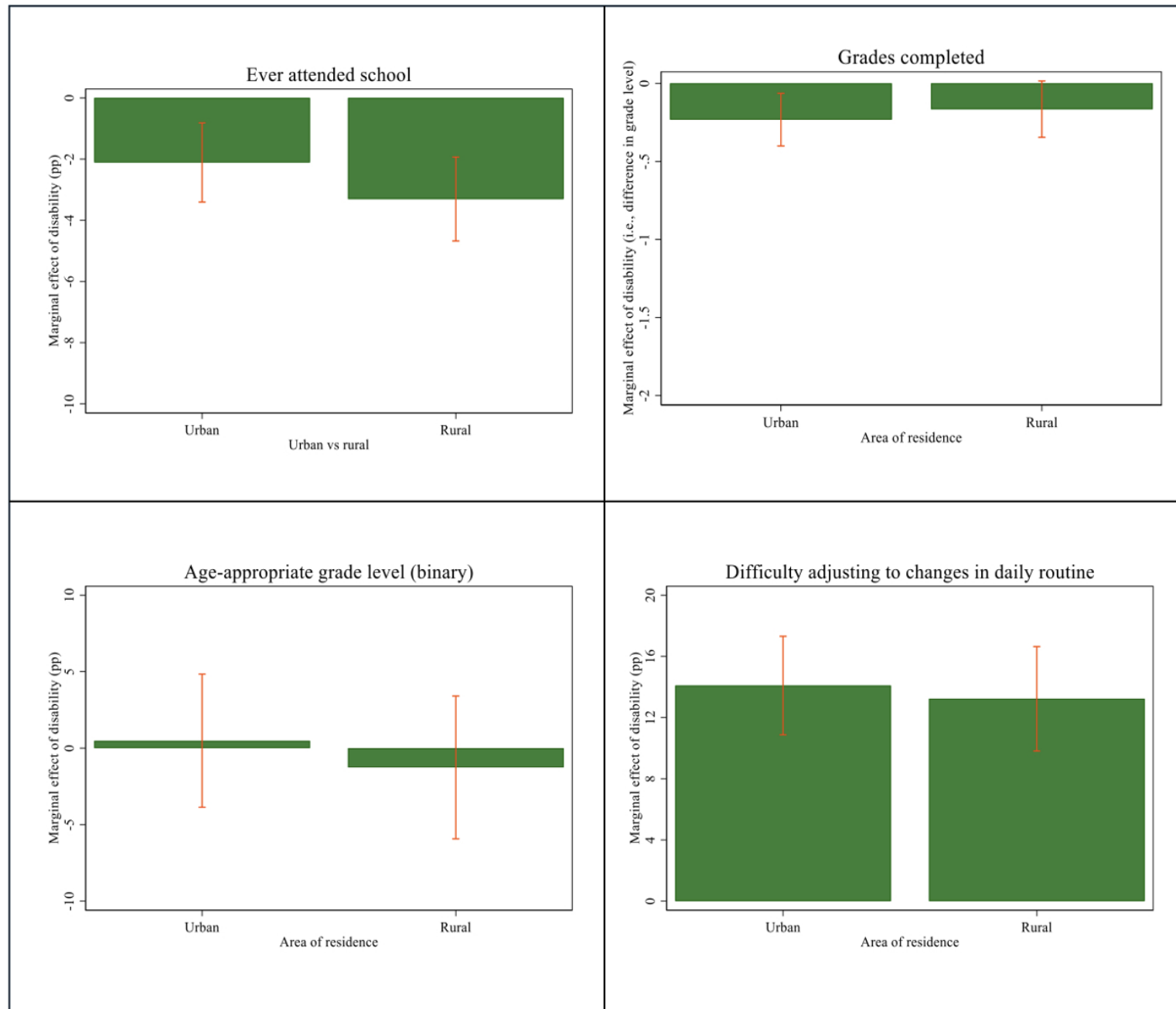

Supplement: S1 Fig — Note: Panel A presents the marginal effects of disability on four educational outcomes by gender, while Panel B presents the corresponding estimates by area of residence (urban versus rural). Outcomes include ever attending school, highest grade attained, age-appropriate grade enrollment, and difficulty adjusting to routine changes. Estimates are derived from linear regression models similar to the main analysis with interaction terms (between disability and the dimension of disadvantage) and adjusted for age group, household characteristics, maternal education, and wealth index. (PDF) [file pgph.0006579.s002.pdf]
