# Supplementary material for: Disability and educational outcomes in Nepal: Evidence from the 2019 Multiple Indicator Cluster Survey
Source: PLOS Glob Public Health. 2026 Jun 4;6(6):e0006579. doi: 10.1371/journal.pgph.0006579 (PMC13235914; doi:10.1371/journal.pgph.0006579)
Supplement: Attachment — Submitted filename: Response to Reviewers.docx [file pgph.0006579.s004.docx]

We would like to sincerely thank the editor and both reviewers for their careful reading of our manuscript and for their constructive and insightful comments. We have revised the manuscript accordingly and believe that the changes have improved its clarity, rigor, and contribution. Below, we respond to each comment point by point. All page and section references refer to the clean version of revised manuscript.

Reviewer 1:

Comment 1 – Abstract: Results – Last sentence: could you clarify what having fewer years of education is referring to… Also the statement “report facing challenges in accepting changes in their daily routine” could use clarification…

Response –

Years of schooling referred to grades completed. We have revised the Results portion of the abstract and mentioned grade completion directly. We have also clarified the part about adapting to changes in daily routines.
These revisions are reflected in the *Results* section under Abstract.

Comment 2 - Methodology: could you provide more detail about who is reporting difficulty with adjusting to routine changes (parent, teacher)?

Response –

We have revised the Methodology section to specify that difficulty adjusting to routine changes is based on reports from the child’s mother or primary caregiver (page 7, para 2).

Comment 3 - Table 1: The percentages with disability and without disability appear almost equal across categories and are confusing…

Response –

Thank you for pointing this out. The percentages shown in the “with disability” and “without disability” columns represent distributions *within each subgroup*, rather than row percentages. To avoid misinterpretation, we have added a clear table note explaining how these percentages should be read.
No changes were made to the underlying data; the clarification appears in the *note below Table 1.*

Comment 4 - Table 2: There is no information about which statistics are being reported… Why report p < 0.10?

Response –

We have revised the note below Table 2 to clearly state that the reported values are regression coefficients, with standard errors shown in parentheses. In addition, we have clarified in the Methodology section that significance levels up to the 10 percent level are reported, following common practice in social science research and in this journal (page 8, para 2).

Reviewer 2:

Comment 1: It would strengthen the manuscript to include a brief contextual overview of Nepal’s education system…

Response –

We appreciate this suggestion. We have added a concise paragraph to the Introduction describing the structure of Nepal’s education system, including the typical age of school entry, compulsory education coverage, and key challenges related to progression and dropout. This addition provides national context for readers unfamiliar with Nepal and helps situate the study’s outcomes (page 6, para 1).

Comment 2: Could you explain what it means to have “difficulty adjusting to routine changes”?

Response –

We clarified in the Methodology that this variable reflects caregiver-reported difficulty adapting to changes in general activities at home (page 7, para 2). We also added a brief clarification in the Discussion when interpreting this outcome (page 15, para 1).

Comment 3: Is there a particular reason why the authors divided the age groups as 5–9, 10–14, and 15–17?

Response –

We have added a justification in the Methodology explaining that these age groups were selected to reflect key educational stages and dropout risks in Nepal, including early primary schooling, transition into lower secondary education, and upper secondary ages when delayed progression and dropout risks increase. Relevant citations have been included. The revised section reads as follows (page 7, para 4):

Age was grouped into 5–9, 10–14, and 15–17 years to reflect key schooling transitions and dropout risks in Nepal. Children 5–9 years are in the early primary grades, while ages 10–14 years span later primary and entry into lower secondary level, when progression starts to diverge. The 15–17 age group corresponds to upper secondary schooling, during which risks of delayed progression and school dropout increase, particularly due to factors such as early marriage.

Comment 4: The study could make a stronger contribution with a more nuanced or heterogeneity analysis…

Response –

We thank the reviewer for this constructive suggestion. In response, we conducted additional heterogeneity analyses to examine whether the association between disability and educational outcomes varies by gender and by area of residence (urban versus rural). The results indicated no statistical differences in the associations across these subgroups. To maintain the focus of the main results on statistically robust findings, we present these analyses in Supporting Information Figure S1 and briefly summarize them in the Results (page 15 para 1) and Discussion sections (page 17 para 2). Overall, the findings suggest that the educational disadvantages associated with disability are broadly consistent across gender and urban–rural contexts.

While these subgroup analyses may have limited power to detect small differences due to smaller sample size, the direction and magnitude of effects were broadly similar across groups.

Comment 5: An alternative approach could be to divide age groups according to compulsory versus post-compulsory education…

Response –

As noted in our response to Comment 3, the age groupings used in this study were selected to reflect key educational stages and transitions in Nepal, including early primary schooling, transition into lower secondary education, and upper secondary ages when risks of delayed progression and dropout increase. While grouping children strictly by compulsory versus post-compulsory education is a valuable alternative framework, the current categorization allows consistent comparison across developmental and schooling stages.

We view analyses explicitly contrasting compulsory and post-compulsory schooling periods as an important direction for future research, particularly with longitudinal data. For now, we have conducted and included a heterogeneity analysis by gender and area of residence, which we believe might be more important for policymaking (please also see response #4).

Thank you again!
