# Supplementary material for: Disability and educational outcomes in Nepal: Evidence from the 2019 Multiple Indicator Cluster Survey
Source: PLOS Glob Public Health. 2026 Jun 4;6(6):e0006579. doi: 10.1371/journal.pgph.0006579 (PMC13235914; doi:10.1371/journal.pgph.0006579)
Supplement: Attachment — Submitted filename: Response_to_Reviewers_auresp_2.docx [file pgph.0006579.s005.docx]

We would like to sincerely thank the editor and reviewers for their careful reading of our manuscript again. We have made the minor revision requested to the manuscript and believe that the change have improved its clarity, rigor, and contribution. Below, we respond to the reviewer’s comment.

Reviewer 2:

Comment 1 – Thank you for thoroughly addressing my earlier comment; the revisions have strengthened the manuscript and improved the clarity of the results. My only remaining suggestion is about the additional results provided in the Supporting Information, which are currently presented as a figure. Given that these results are essentially regression coefficients if I understand correctly, I think it might be more interpretable and useful to readers if the regression coefficients are reported in a standard table format as well.

Response –

Thank you for the suggestion. We have now added a supplementary table reporting the regression coefficients corresponding to the interaction models to improve clarity and interpretability. Table S1 in the Supporting Information section shows the estimated coefficients and standard errors for the interaction terms between disability and gender as well as between disability and area of residence across the four educational outcomes. The table is organized into two panels to separately display the gender and residence interaction models. The previously included marginal effects visualization is also retained as Figure S1 to assist in interpretation of these interaction effects.

Thank you again!
